# Specific arterio-venous transcriptomic and ncRNA-RNA interactions in human umbilical endothelial cells: A meta-analysis

**DOI:** 10.1016/j.isci.2021.102675

**Published:** 2021-05-29

**Authors:** Fabian Vega-Tapia, Estefania Peñaloza, Bernardo J. Krause

**Affiliations:** 1 Instituto de Ciencias de la Salud, Universidad de O'Higgins, Avenida Libertador Bernardo O'Higgins 611, Rancagua, Chile

**Keywords:** Cell biology, Systems biology

## Abstract

Whether arterial-venous differences of primary endothelial cells commonly used for vascular research are preserved *in vitro* remains under debate. To address this issue, a meta-analysis of Affymetrix transcriptomic data sets from human umbilical artery (HUAECs) and vein (HUVEC) endothelial cells was performed. The meta-analysis showed 2,742 transcripts differentially expressed (false discovery rate <0.05), of which 78% were downregulated in HUVECs. Comparisons with RNA-seq data sets showed high levels of agreement and correlation (p < 0.0001), identifying 84 arterial-venous identity markers. Functional analysis revealed enrichment of key vascular processes in HUAECs/HUVECs, including nitric oxide- (NO) and hypoxia-related genes, as well as differences in miRNA- and ncRNA-mRNA interaction profiles. A proof of concept of these findings in primary cells exposed to hypoxia *in vitro* and *in vivo* confirmed the arterial-venous differences in NO-related genes and miRNAs. Altogether, these data defined a cross-platform arterial-venous transcript profile for cultured HUAEC-HUVEC and support a preserved identity involving key vascular pathways post-transcriptionally regulated *in vitro*.

## Highlights

• Transcriptional differences among HUAEC and HUVEC are preserved in culture
• These differences occur even after correcting for experimental conditions
• The heterogenous regulation affects NO- and hypoxia-related genes
• Cell-specific ncRNA/mRNA interactions are found

## Graphical abstract

**Figure:**
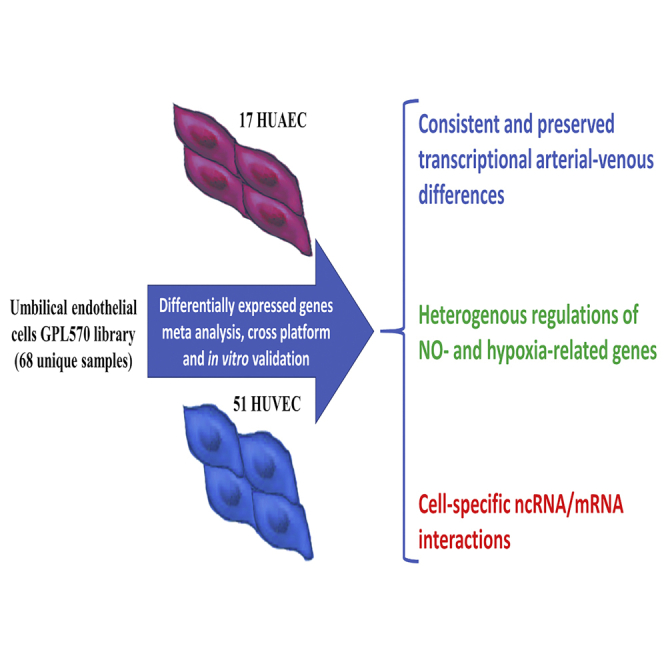

## Introduction

Studies using isolated endothelial cells (ECs) have provided insightful knowledge of vascular physiology because of the remarkable description of cultured human umbilical vein endothelial cells (HUVECs) (Chappey et al., 1996; Onat et al., 2011). Despite the cumulative evidence showing the different susceptibility of arterial and venous ECs to pro-atherogenic and hypertensive factors (Aird, 2015; Chistiakov et al., 2017), several studies consider HUVECs as the model of choice to describe the molecular mechanisms involved in these processes (Richardson et al., 2010). The use of HUVECs to describe arterial and venous EC function might have gained leverage from studies showing that arterial-venous program of ECs is gradually lost *in vitro*, suggesting that endothelial lineage stems from differential adaptation to environmental cues, such as different degrees of shear stress (SS) (Buschmann et al., 2010; Egorova et al., 2012). These observations suggest that EC identity is a meta-state defined by flow dynamics deleted by *in vitro* conditions, favoring HUVECs as a source of universal primary EC instead of arterial alternatives, such as umbilical artery EC (HUAEC), potentially due to the lower yield in culture conditions.

In contrast, studies on early developed blood vessels suggest that long-lasting changes contribute to arterial/venous fate. Expression of ephrin-B2 and Eph-B4 in mouse fetus (Wang et al., 1998) and adult (Gale et al., 2001; Shin et al., 2001) blood vessels is mutually exclusive and is a distinctive trait of arteries and veins, respectively. Similarly, Notch induces ephrin-B2 and inhibits Eph-B4 expression in ECs (Lawson et al., 2001), whereas Nr2f2/COUP-TFII promotes the opposite effect in venous ECs partially mediated by inhibition of Notch signaling (Chen et al., 2012; You et al., 2005), indicating that the arterial and venous transcriptional programs are incompatible. These studies show that arterial and venous ECs have their genetic program that can be induced without dynamic flow-related cues, challenging the concept of SS as a prerequisite for EC differentiation, although these observations alone cannot rebut the loss of arterio-venous characteristics *in vitro*. In this context, the transcriptomic analysis provides valuable information that could solve this controversy. Previous studies comparing the transcriptomic profile between HUAECs and HUVECs have suggested that major differences in the expression of EC differentiation and development occur between freshly isolated cells, including Notch signaling and other arterial/venous determinants, which are lost after 2–3 passages. The expression of many EC phenotype determinants is regulated by epigenetic-based cellular programming; therefore, the differential expression might arise from changes in these regulatory factors induced by culture conditions (Joo et al., 2013; Nakato et al., 2019; Sissaoui et al., 2020). An important issue in the transcriptomic analysis is the limited number of samples within each study (n ≤ 6), which in combination with not well-defined post-test and cutoff values may limit the translational value of these results. Therefore, whether *in vitro* transcriptomic arterial-venous differences between HUAECs and HUVECs are erased during the culturing process affecting the expression of EC differentiation genes (Aranguren et al., 2013; Jiang et al., 2013a, 2013b) or if the effects are even more pervasive remains to be properly addressed. This study aimed to gather transcriptomic data from a broadly used platform to perform a meta-analysis of the transcriptomic profile and differentially expressed genes (DEG) between cultured HUAECs and HUVECs to confirm that arterial-venous identity of cells from a comparable vascular bed is preserved *in vitro*. Results were compared with previous data using this platform and cross-validated with recent RNA-seq data by applying inter-rater agreement tests and other contrast analyses. Finally, based on previous reports from our group, functional enrichment analysis focused on the regulation of NO- and hypoxia-related genes in HUAECs and HUVECs and the potential contribution of non-coding RNA (ncRNA) was assayed to identify post-transcriptional mechanisms regulating arterial-venous differences in these key endothelial pathways. Altogether, this analysis aimed to determine the differences in the artery and venous EC transcriptional program and the impact of the culturing process among commonly used human cell models in cardiovascular research using a meta-analysis approach.

## Results

### Transcriptomic profiling and arterial-venous markers in cultured HUAECs and HUVECs

**Figure 1:**
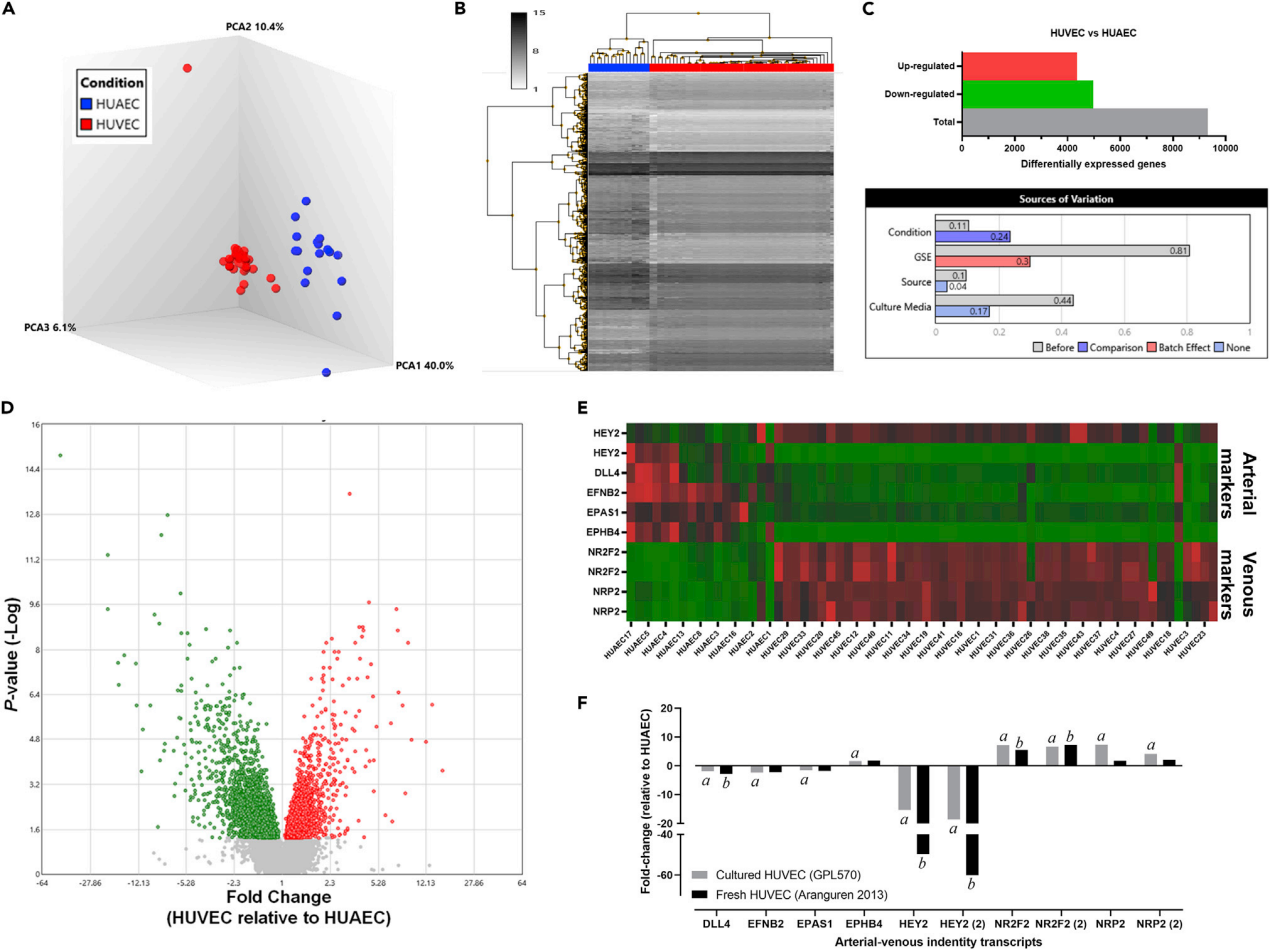
Transcriptomic profiling of human umbilical venous and arterial endothelial cells (A) PCA for the first three components for HUVECs (red) and HUAECs (blue). (B) Unsupervised hierarchical analysis for the top 1,000 DEGs for the HUVEC (red) and HUAEC (blue) data according to gene expression and proximity between samples. The total counts/transcript levels (*Z* score) are indicated in greyscale. (C) Graph bars for the number of both upregulated (red) and downregulated (green) genes in HUVECs versus HUAECs (top) and main sources of variation (bottom). (D) Volcano plot displaying the relative gene expression levels in HUVECs versus HUAECs. Dots indicate significantly enriched transcripts in HUVECs (red) and HUAECs (green). Gray dots represent genes below the significance threshold (FDR-adjusted p value = 0.05, fold change ≥1,5 or ≤ -1,5, FDR ≤0.01). (E) Heatmap for the upregulated (red) and downregulated (green) arterial and venous markers in each interrogated sample. (F) Relative expression of arterial-venous markers in cultured (gray solid bars) and freshly isolated HUVECs (black solid bars) normalized to the average expression in HUAECs. *a*: statistical difference between cultured HUVECs and HUAECs; *b*: statistical difference between freshly isolated HUVECs and HUAECs, p < 0.05.

A comparison of the transcriptomic profile of the GPL570 library showed striking differences of cultured HUVECs and HUAECs according to principal component analysis (PCA) (Figure 1A) and hierarchical clustering (Figure 1B) analyses. A total of 9,329 transcripts were differentially expressed (4,979 downregulated and 4,350 upregulated in HUVECs related to HUAECs, respectively; p < 0.01; false discovery rate [FDR] <0.05), and data set source (i.e., GEO accession number), culture media, and cell type (i.e. HUAEC or HUAEC) (Figure 1C) were the main sources of variation among samples. DEGs were reduced to 2,692 after applying a fold change cutoff of ±1.5 (Figure 1D and Table S1). Analysis of 10 transcripts related to arterial-venous identity showed consistent differential expression between cell types, especially in vein-related markers (Figure 1E). Further comparison of arterial-venous identity transcripts in the GPL570 library (cultured cells) and data from freshly isolated HUAECs and HUVECs reported in the GSE43475 data set confirmed the downregulation of the arterial marker HEY2 in cultured HUVECs (Figure 1F). However, a higher number of vessel identity genes, such as the vein marker NRP2, were differentially expressed in the GPL570 library compared to GSE43475.

### Validation of DEG of the GPL570 library

**Figure 2:**
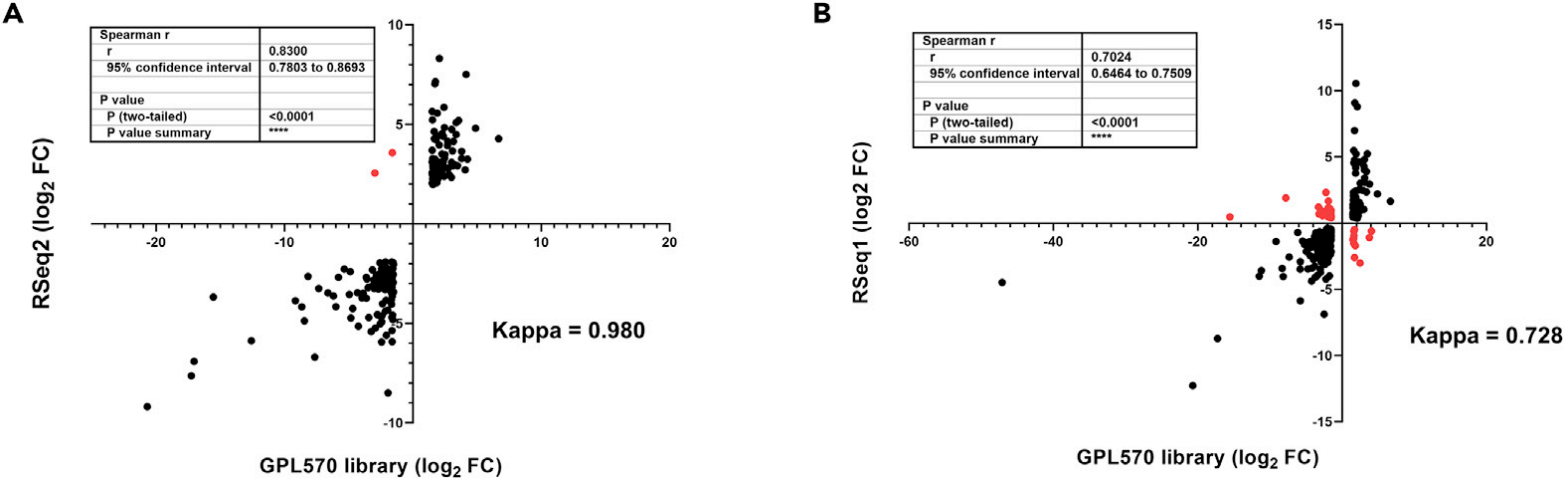
Cross-validation of GPL570 library DEG with RNA-seq data Fold change values of DEG from the GPL570 library were compared with those from the RSeq2 (A) and RSeq1 (B) data sets. Conflicting values (upregulated in one data set and downregulated in the other one or *vice versa*) are shown as red dots. Correlation (inset tables) and inter-rater agreement analysis results (kappa values) are provided.

To validate the obtained transcriptomic profile, the correspondence of these changes was compared with two RNA-seq data sets (RSeq1 and RSeq2). First, fold changes, either increasing or decreasing, were qualitatively compared by the inter-rater agreement test and then were numerically compared by correlation analysis. The sense of the changes in the expression of 205 transcripts (i.e., downregulated or upregulated) described in GPL570 matched those of RSeq2 (203/205) reflected in an almost perfect agreement (Kappa = 0.980), as well as highly significant correlation (r = 0.83, p < 0.0001) (Figure 2A). Comparison of GPL570 to RSeq1 showed a match of 387 transcripts found as DEGs in both data sets, of which 338 showed a fold change sense match that resulted in a substantial agreement (Kappa = 0.728), along with a highly significant correlation (r = 0.7024, p < 0.0001). Considering the compelling agreement and correlation, a list of 84 transcripts that were differentially expressed in the three data sets were identified as potential arterial-venous markers (Table 1).

**Table 1:** Cross-validated differentially expressed genes in HUAEC and HUVEC

DEG	GPL570 library	GSE131681	GSE128382	Average fold-change
HEY2	−20.68	−9.19	−12.27	−13.26
GJA5	−47.11	−6.70	−4.48	−11.22
XG	−17.26	−7.63	−8.72	−10.47
SLITRK4	−11.49	−4.70	−4.00	−6.00
FST	−11.19	−5.24	−3.58	−5.94
RASGRF2	−5.79	−5.93	−5.86	−5.86
PTGS1	2.10	7.50	8.79	5.17
ALDH1A2	1.76	7.15	9.08	4.85
FAM174B	3.50	5.20	5.24	4.57
MAP9	−5.80	−4.65	−3.47	−4.54
LHX6	1.92	4.41	10.55	4.47
SLC46A3	−8.16	−2.64	−4.03	−4.43
ADAMTS18	1.72	7.04	7.00	4.39
ZNF462	3.37	5.09	3.90	4.06
VGLL3	−3.47	−4.57	−3.97	−3.98
RBP1	3.03	4.75	4.28	3.95
CECR2	−7.33	−3.25	−2.56	−3.93
MOCOS	4.22	−2.99	−4.37	−3.81
NR2F2	3.09	3.66	4.80	3.79
TMEM163	−2.48	−3.12	−6.88	−3.76
DNM3OS	4.89	4.80	2.21	3.73
ITGA4	−3.74	−3.27	−4.10	−3.69
SHISA3	−9.15	−3.86	−1.39	−3.66
AR	1.73	5.86	4.77	3.64
MYRIP	6.69	4.29	1.65	3.62
ATP8A1	−5.79	−2.68	−2.91	−3.56
EEF1A2	1.90	5.56	4.19	3.54
ANK3	−4.44	−3.80	−2.45	−3.46
PDE2A	3.80	3.65	2.96	3.45
EPDR1	−3.96	−3.72	−2.73	−3.43
SFRP1	3.16	4.15	3.07	3.43
SLC45A4	−3.80	−3.08	−3.25	−3.36
AUTS2	3.37	4.49	2.35	3.29
FAM107A	−2.99	−3.13	−3.72	−3.26
CXADR	−3.01	−3.25	−3.49	−3.24
HSPB8	3.12	2.92	3.41	3.14
CD44	−2.77	−4.01	−2.78	−3.14
MAMDC2	−4.69	−4.25	−1.51	−3.11
TNFSF15	1.65	3.97	4.55	3.10
PLXNA4	1.93	2.59	5.23	2.97
SLIT2	−4.99	−3.20	−1.48	−2.87
CLU	2.50	3.09	3.01	2.86
FAT1	−2.43	−3.15	−2.89	−2.81
ANTXR1	−3.15	−3.01	−2.29	−2.79
SORT1	−3.65	−2.27	−2.52	−2.75
HIC1	2.27	3.51	2.50	2.71
TSPAN11	2.38	3.16	2.54	2.67
CPXM1	1.87	2.45	3.77	2.58
CAMK2N1	2.37	4.40	1.58	2.54
PDZD2	−3.05	−2.82	−1.87	−2.52
CUBN	−3.58	−2.77	−1.55	−2.49
ITGA1	−1.90	−2.81	−2.59	−2.40
UCP2	1.57	4.23	2.07	2.40
DOK5	1.73	3.93	1.82	2.31
CDC42EP5	1.89	2.96	1.96	2.22
FBP1	−1.62	−3.18	−2.09	−2.21
KLRG1	−1.61	−2.82	−2.36	−2.21
KALRN	1.58	2.82	2.32	2.18
SPHK1	1.86	2.93	1.77	2.13
NRP2	1.68	2.27	2.13	2.01
KANK3	2.47	3.30	0.99	2.01
LGR4	−2.16	−3.31	−1.11	−1.99
INHBA	−3.63	−2.94	−0.73	−1.98
PRR5	1.51	3.70	1.38	1.98
ASAP3	1.61	3.06	1.50	1.95
PALD1	3.01	2.34	1.04	1.94
PTGIS	−2.96	2.55	0.97	−1.94
SORBS2	−3.10	−1.94	−1.14	−1.90
LRP5	2.49	2.82	0.95	1.88
PDE3A	−2.35	−2.20	−1.29	−1.88
ADAMTS7	1.85	2.76	1.30	1.88
TRPV4	1.59	2.38	1.68	1.85
SLCO3A1	−2.56	−1.95	−1.24	−1.84
TUSC3	−3.12	−2.59	−0.65	−1.74
GNA14	−1.5	−2.54	−1.28	−1.71
CAPG	1.94	2.25	1.02	1.64
MMP15	1.54	2.26	1.15	1.59
SLC6A8	1.57	2.23	1.09	1.56
LY75	−1.51	−2.04	−1.21	−1.55
MAP1S	2.24	2.61	0.57	1.49
UBTD1	1.73	2.74	0.69	1.48
WFS1	1.58	2.41	0.64	1.35
MDK	1.73	2.07	0.54	1.25
SH3GL1	1.86	2.07	0.49	1.24

Values expressed as log_(2)_ fold−change in HUVEC relative to HUAEC. Average fold−change calculated as the geometric average of changes in each dataset.

### Expression of key vascular pathways in cultured HUAECs and HUVECs under basal conditions

**Figure 3:**
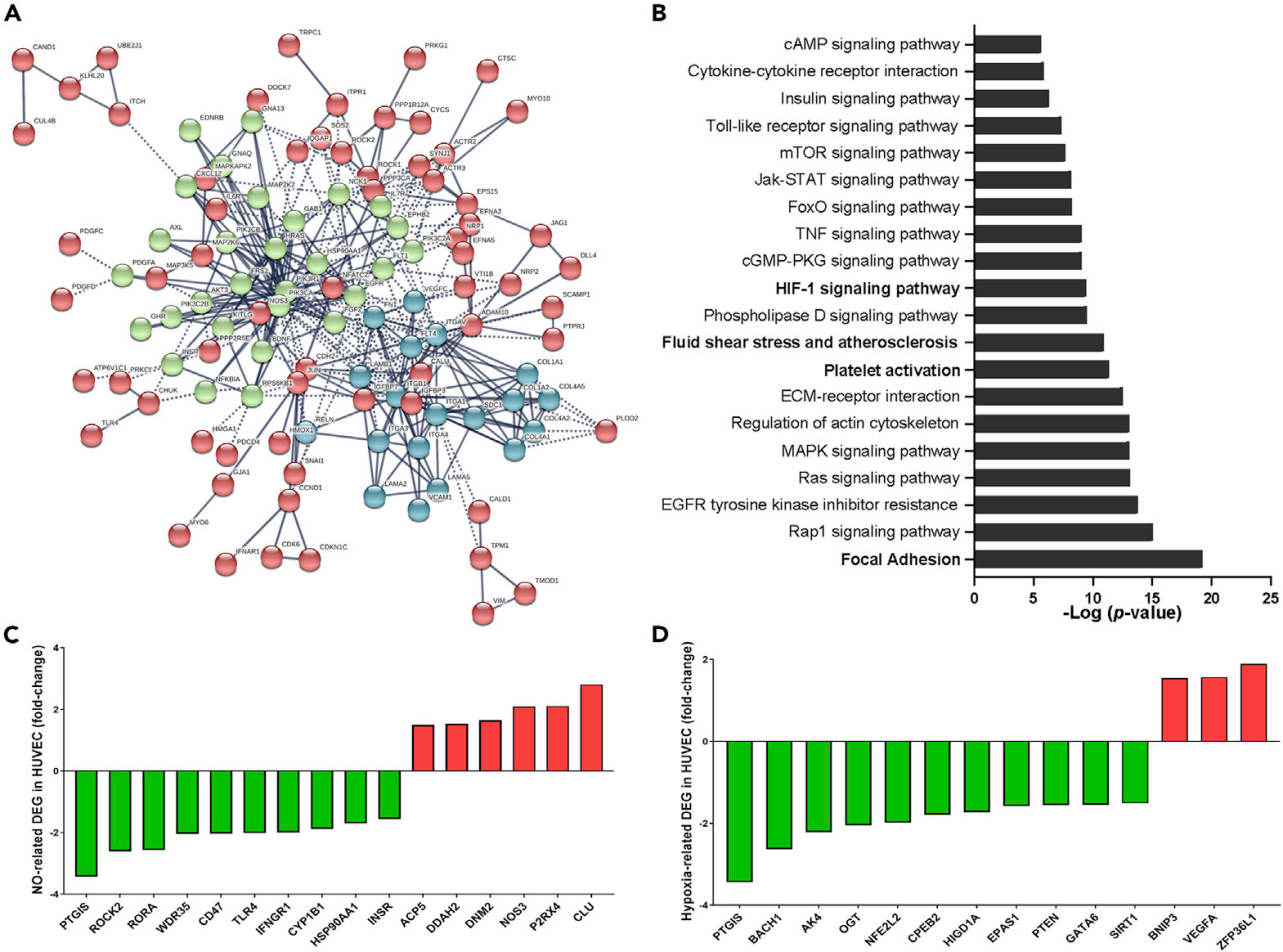
Differential enrichment of pathways related to endothelial function and the differential expression of NO- and hypoxia-related genes in HUVECs and HUAECs (A) Network clustered by k-means (k = 3) based on protein-protein interactions using STRING showing the genes of the top 5 enriched WikiPathways in the gene set of the GPL570 library that are differentially expressed in HUAECs vs HUVECs. Each color represents the cluster the gene belongs to. (B–D) (B) Enriched KEGG pathways for the DEG listed in the top five WikiPathways (adjusted p < 0.05). Key endothelial pathways are highlighted (bold font). Expression values (fold change) for NO- (C) and hypoxia-related (D) genes that are downregulated (green) or upregulated (red) in HUVECs according to the data from the GPL570 library.

Potential functional effects of transcriptional differences between HUAECs and HUVECs were evaluated by selecting genes related to the top five enriched WikiPathways (Slenter et al., 2018), based on the number of associated DEGs. Pathways with a considerable number of DEGs were related to vascular endothelial growth factor (VEGF), miRNA, and PI3K-Akt (Table S2), showing strong interactions among them as suggested by k-means clustering analysis (Figure 3A). These interactions were further confirmed by Kyoto Encyclopedia of Genes and Genomes (KEGG) analysis that resulted in several highly significant pathways (Table S3), with an important representation of key vascular functions, such as focal adhesion, platelet activation, fluid SS, and HIF-1 signaling (Figure 3B and Table 2). Additionally, several genes related to two important endothelial processes (i.e., nitric oxide metabolic process GO:0046209; cellular response to hypoxia GO:0071456) were significantly downregulated in HUVECs compared to HUAECs (Figures 3C and 3D).

**Table 2:** Differentially expressed genes of selected enriched KEGG pathways

Gene	HUVEC R.E. (log2)	HUAEC R.E. (log2)	Fold Change	P-val	FDR P-val	KEGG pathway
AKT3	5.08	6.37	−2.45	0.0026	0.0362	a, b, c, d
COL1A1	4.15	5.35	−2.30	0.0039	0.0442	a, b
COL1A2	5.05	8.87	−14.11	4.15E-5	0.0030	a, b
COL4A1	10.30	12.04	−3.35	0.0002	0.0073	a
COL4A2	11.93	13.26	−2.52	5.91E-6	0.0002	a
EDN1	7.20	8.25	−2.07	0.0006	0.0165	c, d
FLT1	8.85	9.6	−1.68	0.0017	0.0283	a, c
FLT4	6.80	5.93	1.82	0.0032	0.0403	a
GNAQ	8.96	9.56	−1.52	0.0002	0.0079	b
GNAS	6.67	7.5	−1.78	0.0018	0.0292	b
HMOX1	10.22	7.61	6.07	1.78E-7	6.33E-5	c, d
HRAS	9.63	8.71	1.88	0.0003	0.0113	a
IL6R	4.96	4.34	1.54	0.0009	0.0199	d
ITGA1	5.00	5.70	−1.63	1.39E-5	0.0015	a
ITGA3	7.52	8.53	−2.02	8.76E-5	0.0049	a
ITGA4	5.49	7.30	−3.50	0.0002	0.0093	a
ITGAV	11.71	12.74	−2.05	7.43E-7	0.0002	a, c
ITGB3	6.12	5.31	1.76	0.0029	0.0382	a, b, c
ITPR1	6.91	7.51	−1.52	0.0031	0.0393	b
LAMA2	5.44	6.84	−2.64	3.13E-8	1.63E-5	a
MAP2K6	7.37	6.53	1.79	4.90E-6	0.0007	c
MAP3K5	7.27	8.45	−2.26	0.0006	0.0167	c
MEF2A	8.42	9.35	−1.90	0.0024	0.0344	c
MKNK1	5.82	6.78	−1.94	0.0009	0.0205	d
PDGFA	9.01	9.94	−1.90	4.10E-6	0.0006	a, c
PDGFC	9.19	10.83	−3.11	6.67E-6	0.0009	a
PIK3CA	6.08	6.92	−1.79	0.0004	0.0130	a, b, c, d
PIK3CB	8.08	8.73	−1.56	0.0003	0.0115	a, b, c, d
PPP1CA	10.9	10.21	1.64	0.0012	0.0236	a, b
PPP1R12A	9.72	10.34	−1.54	7.25E-5	0.0043	a, b
PRKAA1	7.02	7.75	−1.66	5.40E-6	0.0008	c
PRKCA	4.96	4.25	1.64	0.0031	0.0396	a, d
PRKCI	8.04	8.88	−1.79	0.0003	0.0100	b
PRKG1	5.49	6.38	−1.85	0.0034	0.0412	b
RELN	5.72	7.02	−2.46	0.0004	0.0122	a
SDC1	6.95	6.06	1.85	4.30E-5	0.0031	c
SNAP23	5.60	6.43	−1.79	0.0020	0.0306	b
SOS2	8.66	9.37	−1.63	0.0010	0.0213	a
THBS1	10.68	11.63	−1.93	0.0010	0.0215	a
VCAM1	5.14	9.33	−18.23	4.71E-7	0.0001	c

Lower case letters indicate (a) focal adhesion. (b) platelet activation. (c) fluid shear stress and atherosclerosis and, (d) HIF-1 signaling, from enriched KEGG pathways.

### The potential contribution of ncRNA to the *in vitro* arterial-venous differences

**Figure 4:**
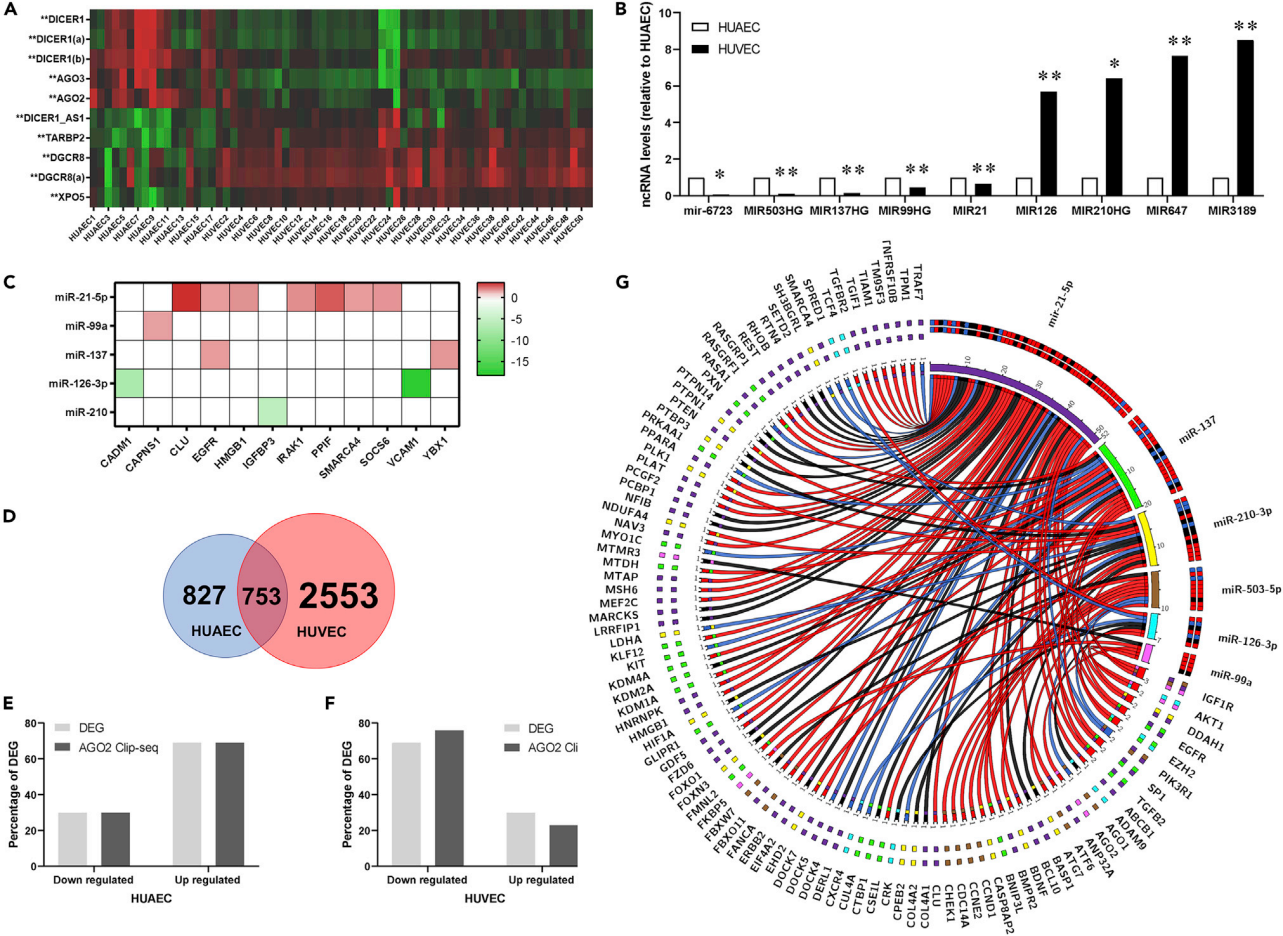
Predicted role of miRNAs in the differential expression of genes in HUAECs and HUVECs (A) Heatmap displaying the expression values of genes involved in miRNA biogenesis and processing from HUAEC (17 leftmost columns) and HUVEC (51 right-most columns) data from the GPL570 library. The transcript expression levels (z-scores) are indicated from the lowest (green) to highest (red). (B) Expression values of miRNA related to endothelial function expressed as fold change (HUVEC vs HUAEC). (C–F) (C) Expression of miRNA targets from the GPL570 data set. MTIs are shown as colored cells with target expression levels as indicated in the color key. Venn diagram showing the overlap between HUAEC and HUVEC transcripts interacting with AGO-2 from AGO2-CLIP-Seq data. Distribution of percentages of DEG in the GPL570 library (light gray bars) and the AGO2-CLIP-Seq data (gray bars) according to gene expression for HUAECs (E) and HUVECs (F). (G) Cord plot displaying MTI for the enriched DE miRNA in the AGO2-CLIP-Seq data from HUAEC and HUVEC (upper right quadrant: miR-21-5p, purple; miR-137, light green; miR-210, yellow; miR-503, brown; miR-126, light blue; miR99a, pink) and DEG from the GPL570 library, and cord color indicates if MTI is specific for HUVEC (red), HUAEC (blue), or not (black). In (B), ∗p < 0.05, ∗∗p < 0.01 relative to HUAEC.

Epigenetic and post-transcriptional mechanisms contribute to arterial-venous differences *in vitro*; thus, we evaluated the participation of miRNAs in the differential expression between HUAECs and HUVECs. Meta-analysis showed that several transcripts implicated in the miRNA-dependent gene regulation (e.g. AGO, DICER1, XPO) (Figure 4A), as well as key miRNAs related to endothelial function (e.g. miR-21, miR-126, and miR-210), were differentially expressed in HUAECs and HUVECs (Figure 4B). Additionally, a limited set of transcripts with high confidence for interaction with these differentially expressed miRNA showed an association between their expression and miRNA levels (Figure 4C).

To further address the role of miRNA, the profile of AGO2-CLIP-seq genes (transcripts found to be bound to AGO2 protein) derived from HUAECs and HUVECs was qualitatively compared. A total of 4,133 transcripts interacting with AGO2 were identified, of which ∼20% were enriched in HUAECs and HUVECs, whereas 2,553 were exclusively detected in HUVECs (Figure 4D). The ratios of downregulated and upregulated genes in the GPL570 library were comparable in the AGO2-CLIP-seq data in HUAECs (Figure 4E) and HUVECs (Figure 4F). However, there was a heterogeneous representation of genes targeted by differentially expressed miRNA between the AGO2-CLIP-seq data from HUAECs and HUVECs (Figure 4G).

### Enriched biological processes related to DEG and AGO2 interactions in HUAECs and HUVECs

**Figure 5:**
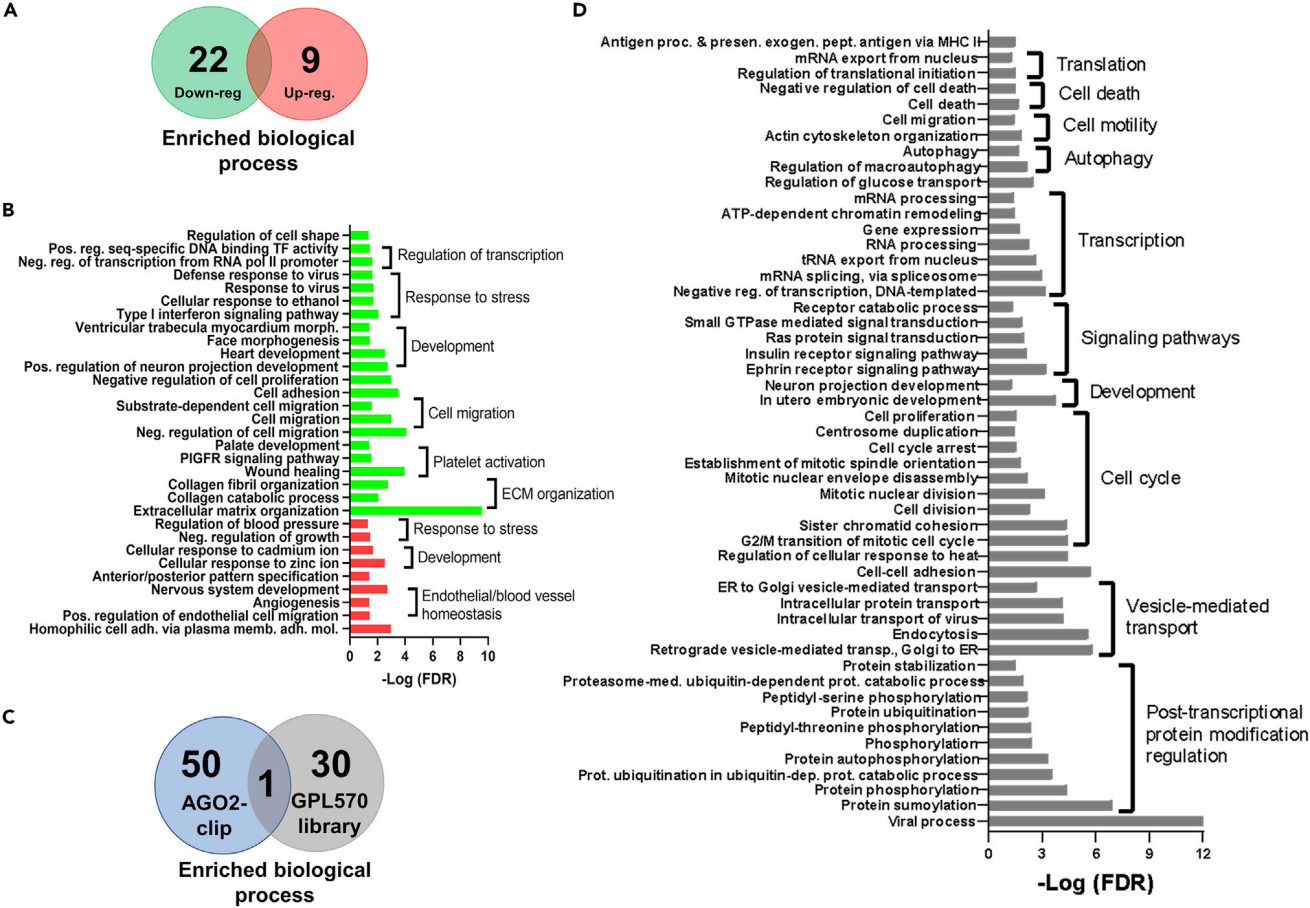
Functional enrichment in HUAECs and HUVECs according to DEG from GPL570 library (A) Venn diagram for the significantly enriched processes in HUVEC samples compared to HUAECs from the GPL570 library. (B) GO terms for biological processes enriched in the downregulated (green) and upregulated (red) DEG subsets in HUVEC vs HUAEC from the GPL570 library. GO terms were grouped according to their relevance to endothelial/vascular function. (C) Venn diagram comparing enrichment of biological process between the GPL570 and the AGO2-CLIP-Seq data sets, listed as the biological process (left list) and are grouped in the common process (right list names) (D) GO terms for biological processes enriched in the differentially expressed miRNA subset in HUVEC vs HUAEC from the AGO2-CLIP-Seq library. GO terms were grouped according to their relevance to cell and developmental function.

To determine which biological processes were differentially enriched in DEG from the GPL570 library and the AGO2-CLIP-Seq data, functional enrichment analysis was performed using the database for annotation, visualization and integrated discovery (DAVID). A total of 31 biological processes were enriched in the DEG from the GPL570 library, and most of them were found in the downregulated gene set from HUVECs (Figure 5A). No match between the enriched processes in the downregulated gene subset (n = 22) and those in the upregulated gene subset (n = 9) was found (Figure 5A). Enriched biological processes (BP) among DEGs showed a considerable relationship with important vascular responses such as extracellular matrix organization, platelet activation, and angiogenesis (Figure 5B). To evaluate potential associations between transcriptional and non-coding RNA-mediated regulation in HUAEC and HUVEC, enriched BP in GLP570 library and arterial-venous differences in AGO2-targeted transcripts were compared. Fifty-one BPs were detected in each gene subsets, with only one common in both data sets (Figure 5C), including cell migration and adhesion, but a poor representation of vascular-specific processes (Figure 5D). Although only one specific enriched process was found in both the GPL570 and AGO2-CLIP libraries, both data sets have enriched processes for the regulation of transcription, cell motility, and cell-cell adhesion.)

### *In vitro* validation of the arterial-venous differences in the control and FGR umbilical endothelial cells

**Figure 6:**
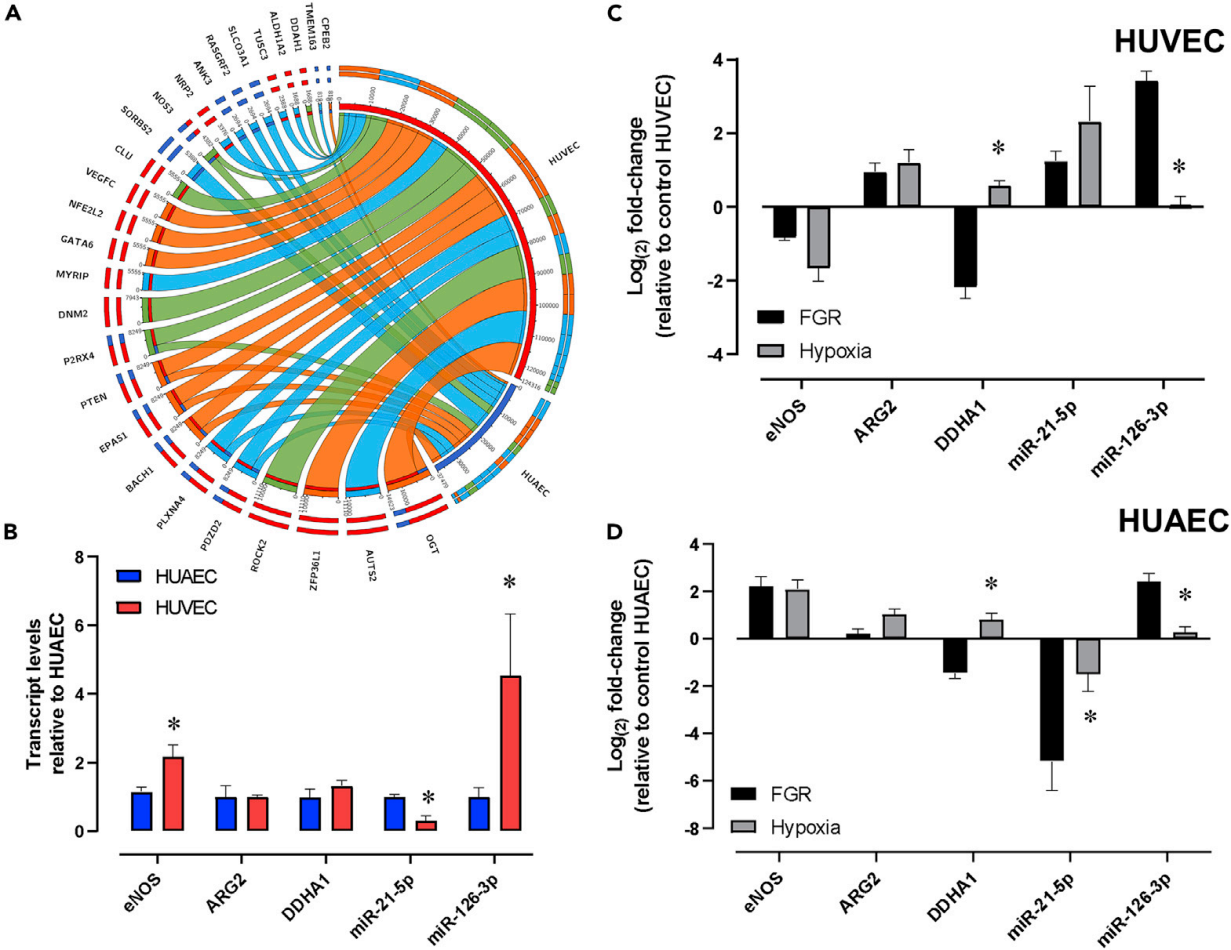
Validation of miRNA and transcript regulation of NO- and hypoxia-related genes in HUAECs and HUVECs (A) Chord plot summarizing the MTI repertoire in HUAECs (blue bands) and HUVECs (red bands) for specific DEG related with NO metabolism (green chords), hypoxia cells response (orange chords), and arterio-venous markers (light blue chords). (B–D) (B) Expression levels for key genes and miRNA involved in the regulation of NO metabolism and hypoxic response in HUVEC vs HUAEC according to the GPL570 library. Expression of NO- and hypoxia-related genes in HUVEC (C) and HUAEC (D) obtained from FGR newborns (black bars) or cultured under hypoxic conditions *in vitro* (gray bars). The expression is expressed as fold change values normalized to the expression in HUAEC cultured under normoxic conditions. In (B), ∗p < 0.05 relative to HUAEC, and in (C) and (D), ∗p < 0.05 relative to FGR cells.

Based on previous reports from our group (Krause et al., 2013; Penaloza et al., 2020), further analyses were focused on the expression of miRNA and transcripts in HUAECs and HUVECs exposed to hypoxia *in vitro* or collected from pregnancies affected by chronic hypoxia (fetal growth restriction, FGR). Data from the AGO2-CLIP-Seq library showed a different repertoire of miRNA-transcript interactions (MTIs) among NO- and hypoxia-related genes in HUAECs and HUVECs (Figure 6A), with a considerable number of MTIs occurring within intronic regions (Table S4). Analysis of the basal expression of eNOS, ARG2, DDAH1, miR-21-5p, and miR-126-3p confirmed a differential regulation, characterized by higher levels of eNOS and miR-126-3p and lower levels of miR-21-5p in HUVECs (Figure 6B). Additionally, umbilical ECs from pregnancies affected by FGR and control cells exposed to hypoxia showed differential regulation of these transcripts and miRNA, resulting in decreased eNOS but increased miR-21-5p levels in HUVECs (Figure 6C). In contrast, eNOS was upregulated in HUAECs exposed to *in vitro* hypoxia and miR-21-5p was downregulated in FGR HUAECs (Figure 6D).

## Discussion

This meta-analysis aimed to comprehensively evaluate the transcriptional profiles of artery and vein ECs from the same vascular beds exposed to static culture conditions and to address differences in the regulation of genes related to key vascular pathways. By gathering sixty-eight transcriptomic data sets from HUAECs and HUVECs, a substantial differentiation for arterial-venous identity was observed in supervised and non-supervised analyses. These profiles showed high consistency and correlation in RNA-seq data, which allowed the identification of 95 transcripts with considerable value for arterial-venous characterization. Notably, significant differences occurred in commonly studied pathways in HUVECs for the characterization of responses to stimulus such as hypoxia, SS, cell adhesion, and platelet activation, among others, occurring in both arteries and veins. Transcriptional changes were also associated with differential expression of highly abundant miRNA in ECs, as well as with differential interacting profiles between mRNA and AGO2. Differences in gene expression were further confirmed by analysis of the expression of NO-related transcripts in HUAECs and HUVECs exposed to hypoxia *in vitro* and cultured cells coming from pregnancies affected by chronic hypoxia. Altogether, these data provide compelling evidence that arterial-venous identity in HUAECs and HUVECs is preserved *in vitro*, affecting several transcripts involved in bed-specific vascular responses, either in basal and pathological conditions.

Primary cultured umbilical ECs have been extensively used for cardiovascular research, mainly due to their availability with very few practical and ethical constraints. HUVECs have been the cornerstone of such studies, making significant advances in the knowledge of diverse physiological and pathological mechanisms (Chappey et al., 1996; Onat et al., 2011), with considerable overrepresentation when compared to other EC types (Richardson et al., 2010). Several studies have demonstrated that umbilical ECs, either HUAECs and HUVECs, express their corresponding arterial-venous genes *in vivo* (Diehl et al., 2005), and it has been suggested that these markers decrease as cells are exposed to culture conditions (Aranguren et al., 2013), an effect also reported in artery ECs from different vascular beds (Burridge and Friedman, 2010). This meta-analysis showed that transcriptomic profiling allows us to clearly differentiate HUAECs and HUVECs by applying non-supervised (hierarchical clustering) and supervised (PCA) analyses, and these differences occurred along with marked differential expression of arterial-venous markers. Accordingly, the effect of culture conditions on transcriptional profiles may not follow a progressive decrease, with fluctuations in diversity among passages (Burridge and Friedman, 2010). Nonetheless, transcriptomic variability was strongly influenced, as may be expected, by batch effect (i.e., source of the data set), but also by the diverse culture media used to generate the data. The evidence shows that basal media, supplementary growth factors, and growth matrix impact on endothelial phenotype, especially as passages increase (Bala et al., 2011; Prasad Chennazhy and Krishnan, 2005; Terramani et al., 2000). Independently of these differences, the high agreement and correlation found with more recent data from RNA-seq and the considerable number of samples collected in this analysis strongly support that arterial-venous differences are substantially maintained *in vitro* (Joo et al., 2013; Nakato et al., 2019; Sissaoui et al., 2020).

Recent reports show that differences between arterial and venous ECs are maintained *in vitro* by epigenetic and post-translational mechanisms that regulated gene expression and responses (Nakato et al., 2019). Direct evidence in HUAECs and HUVECs suggests that these mechanisms modify the chromatin conformation and allow arterial-venous-specific interactions among transcription factors at different promoter locations, with diverse effects (Sissaoui et al., 2020). This study found that transcriptional differences in HUAECs and HUVECs were related to messengers and biological processes contributing to ncRNA-dependent gene regulation, as well as differential expression of key endothelial miRNA (e.g. miR-21-5p and miR-126-3p) (Marin et al., 2013). To further explore the potential participation of ncRNA in this differential expression, AGO2-CLIP-seq data were qualitatively assayed and compared with the profiling obtained from the GLP570 library. The results support the occurrence of specific arterial-venous AGO2-transcript interactions, without a clear association with differentially expressed miRNA between cell types according to the meta-analysis and cross-validation. An original report concerning the AGO2-CLIP data shows that miRNA-transcripts interactions found in HUAECs and HUVECs control the expression of cytoskeletal, contractile, adhesive, and extracellular matrix proteins (Moro et al., 2019), supporting the regulatory role of miRNA. Nonetheless, this meta-analysis showed that several AGO2 transcript interactions also mapped within intronic regions, suggesting additional mechanisms involved in arterial-venous differences. In this regard, circular RNAs (circRNA), spliced from intronic regions of mRNA during transcript processing, have gained attention as a counter-acting mechanism for miRNA transcript targeting, as well as regulators of translation and splicing (Li et al., 2018). Therefore, the consistent presence of AGO2-intronic sequence interactions found here may represent a differential profile of circRNA in HUAECs and HUVECs, as has been reported before (Maass et al., 2017).

Hypoxia and oxygen availability are remarkable stimuli controlling endothelial function at different stages of life and under different pathological conditions (Wong et al., 2017). Several studies concerning cardiovascular function have focused on the regulation by altered oxygen levels of vasoactive pathways, especially the expression of the key endothelial gene eNOS, but the use of ECs from diverse origins has provided controversial evidence. Consistent eNOS downregulation has been reported in HUVECs exposed to *in vitro* hypoxia (Fish et al., 2007, 2010; Janaszak-Jasiecka et al., 2018; Krause et al., 2012, 2013; Phelan and Faller, 1996). In contrast, studies in diverse ECs from arterial origins show eNOS upregulation in response to hypoxia (Justice et al., 2000; Muzaffar et al., 2005; Newman, 1977; Strijdom et al., 2009) including HUAECs (Krause et al., 2012, 2013; Penaloza et al., 2020). Here, we found that transcriptomic differences between HUAECs and HUVECs involved several genes related to NO metabolism and HIF signaling. These differences were also observed at the level of selected genes and miRNAs analyzed in primary cultures of cells exposed to *in vitro* hypoxia, as well as from pregnancies affected by chronic hypoxia. These differences in eNOS expression in normal and pathological conditions have been reported previously by others (Andersen et al., 2009; Jiang et al., 2013a, 2013b; Van de Voorde et al., 1987). Additionally, previous studies from our group have shown the contribution of gene-specific epigenetic modifications in regulating eNOS in HUAECs and HUVECs under normoxia and hypoxia, both *in vivo* and *in vitro* (Krause et al., 2013, 2016). Additionally, recent reports show that miRNAs that are regulated by hypoxia, such as miR-21-5p (Penaloza et al., 2020) and miR-200b (Janaszak-Jasiecka et al., 2018), participating in the regulation of NO-related genes in hypoxia. Based on this evidence, it is possible to suggest the preferential use of HUAECs for studies addressing the regulation of NO-related genes by hypoxia in arterial circuits, improving the translational value of *in vitro* studies.

Altogether, this study provides convincing evidence concerning the transcriptomic profiles for HUAECs and HUVECs under basal conditions and the differences in the expression of key vascular pathways commonly studied in these *in vitro* models of endothelial function, such as a major endothelial-dependent vasodilator pathway (i.e., nitric oxide). These observations raise concerns on the use of HUVECs as an overarching model of human ECs; therefore, the use of HUAECs is urged for the study of arterial EC function. These differential profiles comprise dozens of genes that may be applied for arterial-venous characterization due to their presence in diverse transcriptomic analysis platforms. Preliminary analysis suggests that transcriptomic differences may affect ncRNA; thus, further studies addressing the role of miRNA and circRNA would contribute to confirm their role in maintaining arterial identities *in vitro*.

### Limitations of the study

This article aimed to consolidate, by performing a meta-analysis, a transcriptional profile related to *in vitro* differences between HUAECs and HUVECs. Based on the data, it is possible to argue that cultured umbilical ECs preserve some considerable differences related to their vascular bed of origin; however, if these differences are only restricted to the arterial-venous identity, it requires further analysis. Conversely, validation studies were performed at comparable passages of the data set studies but using a defined culture media that are known to affect the phenotype. Moreover, the significance of these differences needs to be addressed considering the stage of development at which they are obtained (i.e. neonatal age) and including the exposure to simulated SS to compare the role of that factor.

## STAR★Methods

### Key resources table

**Table:** 

REAGENT or RESOURCE	SOURCE	IDENTIFIER
**Biological Samples**		

Primary cultures of HUVEC from Placenta	Faculty of Medicine at the Pontifical Universidad Catholica of Chile	Protocol number 170705023
Primary cultures of HUAEC from Placenta	Faculty of Medicine at the Pontifical Universidad Catholica of Chile	Protocol number 170705023

**Chemicals, Peptides, and Recombinant Proteins**		

TRizol reagent	Invitrogen	15596018

**Critical Commercial Assays**		

MystiCq®microRNA cDNA Synthesis Mix	Sigma-Aldrich	MIRRT
MystiCq® microRNA® SYBR® Green qPCR ReadyMix™	Sigma-Aldrich	MIRRM00
OneScript® Plus cDNA Synthesis Kit	ABM	G236
KiCqStart® SYBR® Green qPCR ReadyMix™	Sigma-Aldrich	KCQS02

**Deposited Data**		

Gene Expression Omnibus repository	GEO; https://www.ncbi.nlm.nih.gov/geo/	#GPL570

**Oligonucleotides**		

miR-21-5p	Sigma-Aldrich	MIRAP00047
miR-126-3p	Sigma-Aldrich	MIRAP00141
eNOS	IDT	N/A
DDAH1	IDT	N/A
ARG2	IDT	N/A
SOD1	IDT	N/A
ATPSF1	IDT	N/A
RPLP2	IDT	N/A
MystiCq® Universal PCR Primer	Sigma-Aldrich	MIRUP

**Software and Algorithms**		

Transcriptome Analysis Console 4.0.2.15	Applied Biosystems	https://www.thermofisher.com/cl/es/home/life-science/microarray-analysis/microarray-analysis-instruments-software-services/microarray-analysis-software/affymetrix-transcriptome-analysis-console-software.html
QuickCalcs GraphPad	GraphPad	https://www.graphpad.com/quickcalcs/kappa1.cfm
miRTarBase	Huang HY, Lin YC, Li J, Huang KY, Shrestha S, Hong HC, Tang Y, Chen YG, Jin CN, Yu Y, Xu JT, Li YM, Cai XX, Zhou ZY, Chen XH, Pei YY, Hu L, Su JJ, Cui SD, Wang F, Xie YY, Ding SY, Luo MF, Chou CH, Chang NW, Chen KW, Cheng YH, Wan XH, Hsu WL, Lee TY, Wei FX, Huang HD^∗^. (2020) Nucleic Acids Research.	http://mirtarbase.cuhk.edu.cn/php/index.php
Table Browser of the Genome Browser	Karolchik D, Hinrichs AS, Furey TS, Roskin KM, Sugnet CW, Haussler D, Kent WJ. The UCSC Table Browser data retrieval tool.*Nucleic Acids Res*. 2004 Jan 1;32(Database issue):D493-6.	https://genome.ucsc.edu/cgi-bin/hgTables
DAVID v6.8	Huang da W, Sherman BT, Lempicki RA. Bioinformatics enrichment tools: paths toward the comprehensive functional analysis of large gene lists. Nucleic Acids Res. 2009 Jan;37(1):1-13. https://doi.org/10.1093/nar/gkn923. Epub 2008 Nov 25. PMID: 19033363; PMCID: PMC2615629. Huang da W, Sherman BT, Lempicki RA. Systematic and integrative analysis of large gene lists using DAVID bioinformatics resources. Nat Protoc. 2009;4(1):44-57. https://doi.org/10.1038/nprot.2008.211. PMID: 19131956.	http://david.abcc.ncifcrf.gov/
Cytoscape v3.8.0.	Shannon P, Markiel A, Ozier O, Baliga NS, Wang JT, Ramage D, Amin N, Schwikowski B, Ideker T. Cytoscape: a software environment for integrated models of biomolecular interaction networks. Genome Res. 2003 Nov;13(11):2498-504. https://doi.org/10.1101/gr.1239303. PMID: 14597658; PMCID: PMC403769.	https://cytoscape.org/download.html
String 11.0	Szklarczyk D, Gable AL, Lyon D, Junge A, Wyder S, Huerta-Cepas J, Simonovic M, Doncheva NT, Morris JH, Bork P, Jensen LJ, Mering CV. STRING v11: protein-protein association networks with increased coverage, supporting functional discovery in genome-wide experimental data sets. Nucleic Acids Res. 2019 Jan 8;47(D1):D607-D613. https://doi.org/10.1093/nar/gky1131. PMID: 30476243; PMCID: PMC6323986.	https://string-db.org/)
GraphPad Prism 8	GraphPad Software Inc., CA	https://www.graphpad.com/scientific-software/prism/
Affymetrix GeneChip HG-U133 Plus 2.0 platform	Thermo Fisher Scientific	http://www.affymetrix.com/support/technical/byproduct.affx?product=hg-u133-plus

### Resource availability

#### Lead contact

Further information and requests for resources and reagents should be directed to and will be fulfilled by the lead contact, Dr. Bernardo J. Krause (bernardo.krause@uoh.cl/bjkrause@gmail.com).

#### Materials availability

This study did not generate new unique reagents.

#### Data and code availability

The published article includes all data sets generated during this study, meanwhile the original data sources are available in the Gene Expression Omnibus (data sets codes described in Table S5).

### Experimental model and subjects details

Ethics approval was obtained for studies involving primary cultures of HUVEC and HUAEC, by the corresponding ethics committees of the Faculty of Medicine at the Pontifical Universidad Catholica of Chile (Protocol number 170705023. Written informed consent was obtained from all those mothers who agreed to participate before collecting any samples or clinical/demographic data, following inclusion criteria previously described (Krause et al., 2013; Penaloza et al., 2020).

### Methods details

#### PCR reactions

**Table:** miRNA-RT Poly(A) Tailing Reaction

Reagent	1X	Amount
Poly(A) Tailing Buffer	5X	2 μL
RNA	1 μg	7° - μL
RNase-free water	n/a	7 – volume RNA
Poly A Polymerase		1 μL
**Total**	**n/a**	10 μL

**Table:** miRNA-RT First-strand cDNA Synthesis Reaction

Reagent	1X	Amount
Poly (A) Tailing Reaction	n/a	10 μL
MystiCq cDNA Reaction Mix		9 μL
ReadyScript Reverse Transcriptase		1 μL
**Total**	**n/a**	20 μL

**Table:** miRNA qPCR

Reagent	1X	Amount
MystiCq microRNA SYBR Green qPCR ReadyMix	2X	5μL
MystiCq Universal PCR Primer	10 uM	0.2 μL
MystiCq microRNA Assay Primer	10 uM	0.2 μL
RNase free water	n/a	2.6 μL
cDNA	0,1 ng – 10 ng	2 μL
**Total**	**n/a**	10 μL

##### Meta-analysis

A specific platform and assay version was selected (Affymetrix GeneChip HG-U133 Plus 2.0 platform) based on the data set availability in the Gene Expression Omnibus repository (GEO; https://www.ncbi.nlm.nih.gov/geo/) (platform accession #GPL570). Data sets were browsed applying the guidelines from Prisma Equator for meta-analysis studies (https://www.equator-network.org/reporting-guidelines/prisma/) detailed in Figure S1. The browsing process was performed using the keywords “umbilical vein”, “umbilical artery” and “HUVEC”, in combination with “GPL570” to exclude data generated with other assays, and metadata, including cell source and culture conditions, was obtained for further analysis. The search considered all data available up to November 2020, retrieving a total of 82 data sets of which 35 met the follow inclusion criteria:

a. Cells cultured for 1 – 5 passages
b. Cells cultures under standard conditions (21% O_2_, 5% CO_2_)
c. No treatment, vehicle or mock manipulation
d. Raw CEL file availability

A total of 68 unique samples (HUAEC = 17; HUVEC = 51), named as GPL570 library for this study (Table S5). Data contained in CEL files were directly analyzed using the software Transcriptome Analysis Console 4.0.2.15 (Applied Biosystems). Analysis parameters considered the following cutoff values: -1.5 < fold change > 1.5; a p-value < 0.01 and FDR < 0.05 using the Limma with empirical Bayes method for multiple comparison. DEGs in HUAEC and HUVEC, and the top five Wiki-Pathway for differentially expressed (DE) genes between cultured HUAEC and HUVEC were derived from this assay for further analysis. Comparison with data from freshly isolated HUAEC and HUVEC(Aranguren et al., 2013) was performed by applying the analysis strategy described to the GSE43475 data set.

##### Cross-Validation

To determine the concordance between the DEG in the GPL570 library with RNA-seq profiling data for HUAEC and HUVEC, DEG from GSE128382 (RSeq1) and GSE131681 (RSeq2) data sets were compared by determining the inter-rater agreement Kappa-value. Briefly, DEG were filtered according to their presence, regardless of the magnitude and sense of fold-change (down/up-regulation), in the GPL570 library and the corresponding data set. Then the agreement in the determination of down-regulated or up-regulated genes was estimated using the webtool QuickCalcs from GraphPad (https://www.graphpad.com/quickcalcs/kappa1.cfm). Additionally, Spearman correlation analysis was performed for each RNA-seq data set relative to the GPL570 library. Cutoff criteria for agreement and correlation were a Kappa-value < 0.700 (substantial agreement) and p-value < 0.01, respectively.

##### Predicted role of miRNAs

Experimentally validated human miRNA-target interaction (MTI) data was obtained from miRTarBase (http://mirtarbase.cuhk.edu.cn/php/index.php, last updated June 30, 2019), which offers curated MTI data and classifies the information as strong (reporter assay, western blot, qPCR) or less strong evidence (microarray, NGS, other methods). MTIs with strong supporting data were selected and a new database was generated and cross-referenced with a list of differentially expressed miRNAs from the GPL570 library to determine potential differentially expressed miRNA targets. To determine the regulation of DEG in HUAEC and HUVEC, data sets from Argonaute-2 (AGO-2) RNA-seq experiments were analyzed (GSE99686). Briefly, cells in the third passage were treated with UV-light to cross-link Argonaute-2/miRNA/transcript complexes, which were subsequently isolated by immunoprecipitation with an antibody against AGO-2A8, and the nature of the transcripts was determined by RNA-seq profiling. Based on CIMS analysis data(Bottini et al., 2017; Moore et al., 2014), transcript peaks with high confidence (FDR < 0.01) belonging to the 3 upper quartiles of counts (>800 counts) were selected and mapped using the webtool Table Browser of the Genome Browser (https://genome.ucsc.edu/cgi-bin/hgTables) with the genome build hg19. Representation of DEG from the GPL570 library among AGO-2-interacting transcripts was qualitatively assayed by Venn diagrams, with a focus on arterial-venous-specific transcripts and genes related to nitric oxide and hypoxia pathways.

##### Functional enrichment analysis of DEG

To address the role of the DEGs and AGO-2-transcripts interaction between HUAEC and HUVEC functional enrichment was performed. For DEGs, genes that had transcript variants with changes in opposite directions (at least 1 upregulated and at least 1 downregulated variant) were removed (3 genes). Two lists from the selected HUVEC DEGs were generated, one for upregulated DEGs and one for downregulated DEGs, and submitted to functional analysis using DAVID v6.8 (REF: PMID: 19033363, PMID: 19131956) to obtain a list of enriched biological processes (GO Terms) in both lists. Network analysis was performed for both lists using Cytoscape v3.8.0.

##### Potential protein-protein interaction networks functional enrichment analysis

Based on the top five Wiki-Pathway for the DEGs from the GPL570 library, functional analysis of potential protein-protein interactions was performed using String 11.0 (https://string-db.org/). The analysis was performed using a high confidence index (≥ 0.900), considering experiment, databases, co-expression, neighborhood, and co-occurrence as sources of evidence of protein-protein interaction. Functional clustering was performed using the K-means algorithm for 3 clusters, and functional enrichment of KEGG pathways was derived from this set of data.

##### Validation of miRNA and transcript regulation of NO- and hypoxia-related genes in HUAEC and HUVEC

HUVEC and HUAEC were isolated by collagenase digestion from umbilical cords samples. Cells isolated from control and FGR placenta were cultured in MCBD131 medium (SIGMA) with Microvascular Growth Supplement (MVGS, S00525, Invitrogen Waltham, Massachusetts, USA). Upon confluence, cells were plated up to the second or third passage and then serum-starved (2% MVGS). Control ECs were exposed to normoxia or hypoxia (1% oxygen) in a chamber (Proox 110, Biospherix) for 0, 6, and 48 hours (Krause et al., 2013).

Total RNA was isolated using a standard TRIzol protocol (Thermo Fisher User Guide - Pub. no. MAN0016385 - Rev. A.0 / 4) according to the manufacturer’s instructions skipping the last wash to minimize small RNA loss. RNA concentrations were calculated based on the absorbance at 260 nm measure with the Nanodrop system (Thermofisher Nanodrop TM) (Penaloza et al., 2020).

The cDNA synthesis for qPCR was generated with MystiCq® microRNA cDNA Synthesis Mix (Sigma Aldrich) for miRNA and OneScript cDNA Synthesis Kit (ABM) for mRNA. Levels of both miRNA and mRNA were quantified through quantitative real-time PCR (qRT-PCR) in a StepOne Plus system (Applied Biosystems), using KiCqStart Kit (Sigma Aldrich https://www.sigmaaldrich.com/technical-documents/protocols/biology/kicqstart-sybr.html) for mRNA and MystiCq kit (Sigma Aldrich https://www.sigmaaldrich.com/technical-documents/protocols/biology/mysticq-microrna-analysis.html) for miRNAs. A list of the primers and their sequence is given in Table S6. All procedures were realized according to the manufacturer’s protocol. The relative expressions were calculated using the 2^−ΔΔCt^ method; the numeric results represent the geometric average of the relative expression determined with 2 housekeeper genes (Penaloza et al., 2020).

**Table:** PCR conditions (mRNA)

PCR cycling conditions			
Steps	Temperature	Time	Cycles
Initial Denaturation	95°C	03 min	1
Denaturation	95°C	05 sec	40 cycles
Annealing	58°C	15 sec	
Extension	°C	15 sec	
Final extension	72°C	5 min	1
Hold	4°C	Forever

**Table:** PCR conditions (miRNA)

PCR cycling conditions			
Steps	Temperature	Time	Cycles
Initial Denaturation	95°C	03 min	1
Denaturation	95°C	05 sec	40 cycles
Annealing	60°C	15 sec	
Extension	70°C	15 sec	
Final extension	72°C	5 min	1
Hold	4°C	Forever	

### Quantification and statistical analysis

All values were expressed as mean ± standard error of the mean (SEM). Comparisons between the two groups were performed by non-parametric Mann-Whitney U-test. For the statistical analysis of hypoxia-treatment and comparisons for three groups or more, one-way ANOVA, or two-way ANOVA was performed as appropriate. If the ANOVA demonstrated a significant interaction between variables, *post hoc* analysis was performed by a false discovery rate (FDR) method. In all the analyses, nominal Mann-Whitney or FDR-adjusted (ANOVA) p < 0.05 was considered for the determination of statistical significance. All statistical analysis was conducted using GraphPad Prism 8 (GraphPad Software Inc., CA).

## Supplemental information

Document S1. Figure S1 and Tables S4 and S6

Table S1. Detailed list of DEG in HUVEC respect HUAEC. Descriptive list including all differentially expressed genes with > +/−1.5 FC, shown p-value, FDR, and both adjusted, Related to main Figure 1

Table S2. List of DEG included in top-five Wikipathways. Detailed list show pathways that contain more DEG, up and down-regulation proportion, significance, and p-value, Related to main Figure 3

Table S3. List of KEGG pathways enrichment. Descriptive list of KEGG Term, along with observed gen count, background gene count, FDR, and matching proteins in the network (IDs and labels), Related to main Figure 3

Table S5. Summary of data available details and inclusion/exclusion criteria, Related to STAR Methods Meta-analysis
