# Supplementary material for: Specific arterio-venous transcriptomic and ncRNA-RNA interactions in human umbilical endothelial cells: A meta-analysis
Source: iScience. 2021 May 29;24(6):102675. doi: 10.1016/j.isci.2021.102675 (PMC8243012; doi:10.1016/j.isci.2021.102675)
Supplement: Document S1. Figure S1 and Tables S4 and S6 [file mmc1.pdf]

**iScience, Volume *24***

## Supplemental information

# Specific arterio-venous transcriptomic and ncRNA-RNA interactions in human umbilical endothelial cells: A meta-analysis

**Fabian Vega-Tapia, Estefania Peñaloza, and Bernardo J. Krause**

**Figure S1**: Flowchart used for meta-analysis based on Prisma Equator guidelines. Related to STAR Methods Meta-analysis

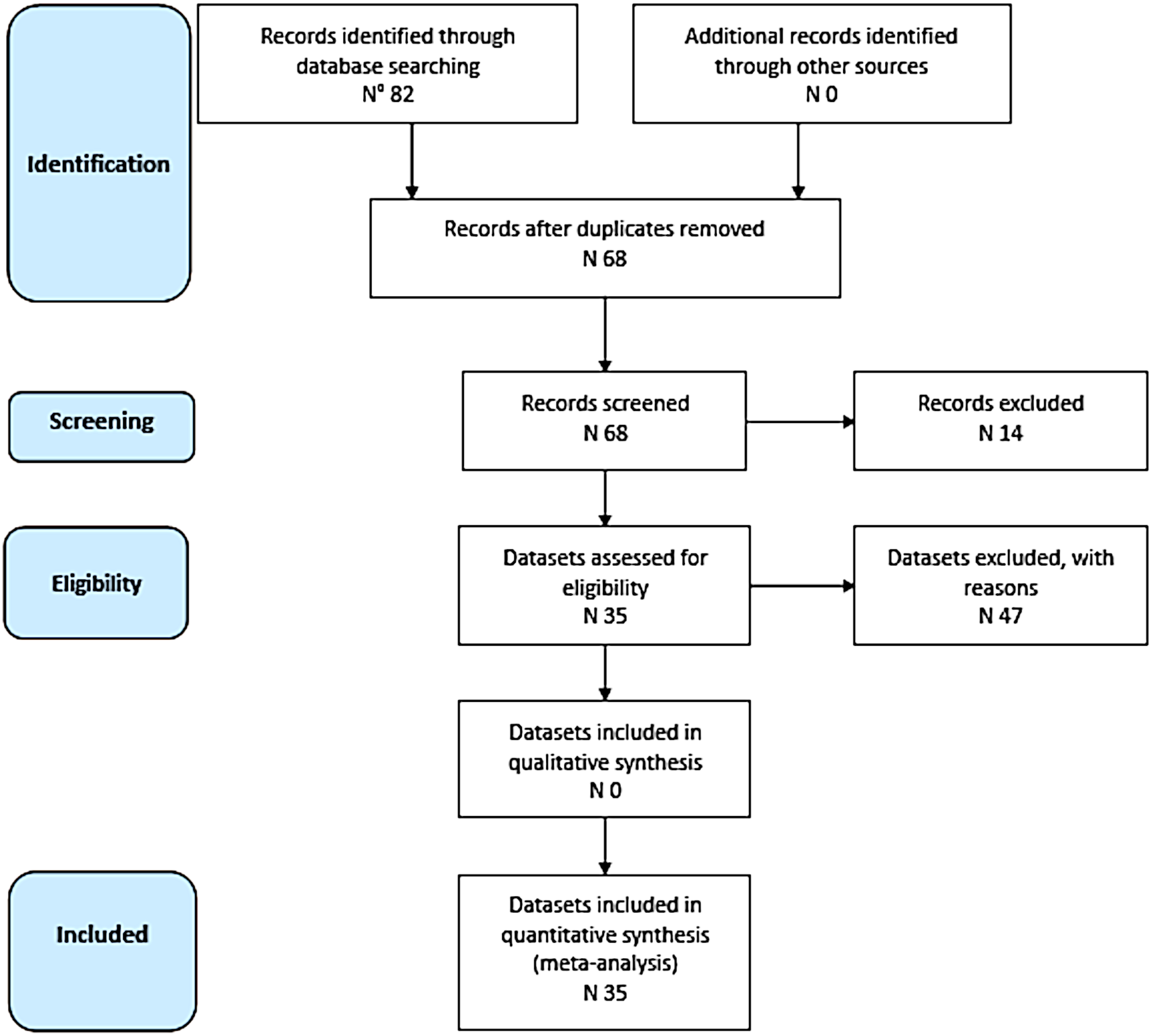

**Table S4: Summary of NO- and hypoxia-related genes targeted in AGO2-Clip data.**
**Related to main Figure 6**
**HUAEC**

| AGO2-clip data | | | | | | Targeted-gene info | | | | | | |
|---|---|---|---|---|---|---|---|---|---|---|---|---|
| **Chromosome** | **Strand** | **chromStart** | **chromEnd** | **FDR** | **count(>=m,k)** | **Gene symbol** | **Gene Start** | **Gene End** | **Ensembl ID** | **Association** | **Transcript variants** | **Interacting region** |
| chr10 | - | 62158807 | 62158808 | 0.004083148 | 2694 | ANK3 | 61786055 | 62332714 | ENSG00000151150 | A-V marker | 205 | Intron 2-3 |
| chr10 | - | 62158807 | 62158808 | 0.004083148 | 2694 | ANK3 | 61786055 | 62493284 | ENSG00000151150 | A-V marker | 216 | Intron 1-2 |
| chr10 | - | 62158807 | 62158808 | 0.004083148 | 2694 | ANK3 | 61843696 | 62332714 | ENSG00000151150 | A-V marker | 232 | Intron 1-2 |
| chr21 | + | 30702386 | 30702387 | 0.004083148 | 2694 | BACH1 | 30671219 | 30718469 | ENSG00000156273 | HIF | 201, 202 | Intron 4-5 |
| chr21 | + | 30702386 | 30702387 | 0.004083148 | 2694 | BACH1 | 30671736 | 30718469 | ENSG00000156273 | HIF | 203 | Intron 2-3 |
| chr21 | + | 30702386 | 30702387 | 0.004083148 | 2694 | BACH1 | 30677559 | 30734217 | ENSG00000156273 | HIF | 208 | Intron 2-3 |
| chr4 | + | 15069275 | 15069276 | 0.004083148 | 819 | CPEB2 | 15004297 | 15071777 | ENSG00000137449 | HIF | 202,204; 203; 208 | Exons 11; 10; 12 |
| chr2 | + | 46613532 | 46613533 | 0.004083148 | 2694 | EPAS1 | 46524540 | 46613842 | ENSG00000116016 | HIF | 201 | Exon 16 |
| chr2 | + | 46613532 | 46613533 | 0.004083148 | 2694 | EPAS1 | 46606838 | 46613842 | ENSG00000116016 | HIF | 206 | Exon 5 |
| chr7 | + | 150700898 | 150700899 | 0.004083148 | 2694 | NOS3 | 150688143 | 150711687 | ENSG00000164867 | NO | 201 | Intron 14-15 |
| chr7 | + | 150700898 | 150700899 | 0.004083148 | 2694 | NOS3 | 150690840 | 150701023 | ENSG00000164867 | NO | 204, 210 | Exon 14 |
| chrX | + | 70756278 | 70756279 | 0.004083148 | 2694 | OGT | 70752911 | 70795747 | ENSG00000147162 | HIF | 201 | Intron 2-3 |
| chrX | + | 70794301 | 70794302 | 0.004083148 | 819 | OGT | 70752911 | 70795747 | ENSG00000147162 | HIF | 202 | Exon 22 |
| chrX | + | 70794301 | 70794302 | 0.004083148 | 819 | OGT | 70767070 | 70795747 | ENSG00000147162 | HIF | 210 | Exon 20 |
| chrX | + | 70756278 | 70756279 | 0.004083148 | 2694 | OGT | 70752911 | 70765701 | ENSG00000147162 | HIF | 202 | Intron 2-3 |
| chr12 | + | 121650388 | 121650389 | 0.004083148 | 2694 | P2RX4 | 121647663 | 121670479 | ENSG00000135124 | NO | 202,203,205,206,208, 210-213 | Intron 1-2 |
| chr12 | + | 121650388 | 121650389 | 0.004083148 | 2694 | P2RX4 | 121647663 | 121671909 | ENSG00000135124 | NO | 207, 209, 214 | Intron 1-2 |
| chr5 | + | 32061205 | 32061206 | 0.004083148 | 2694 | PDZD2 | 31639516 | 32111038 | ENSG00000133401 | A-V marker | 202 | Exon 14 |
| chr5 | + | 32061205 | 32061206 | 0.004083148 | 2694 | PDZD2 | 31798995 | 32111038 | ENSG00000133401 | A-V marker | 203 | Exon 13 |
| chr5 | + | 32061205 | 32061206 | 0.004083148 | 2694 | PDZD2 | 31855042 | 32078061 | ENSG00000133401 | A-V marker | 208 | Exon 4 |
| chr7 | - | 132158978 | 132158979 | 0.004083148 | 2694 | PLXNA4 | 131808090 | 132261323 | ENSG00000221866 | A-V marker | 201, 202 | Intron 3-4 |
| chr7 | - | 132158978 | 132158979 | 0.004083148 | 2694 | PLXNA4 | 132068246 | 132261323 | ENSG00000221866 | A-V marker | 204 | Intron 3-4 |
| chr10 | + | 89630010 | 89630011 | 0.004083148 | 2694 | PTEN | 89623194 | 89728532 | ENSG00000171862 | HIF | 201, 202 | Intron 1-2 |
| chr5 | + | 80292226 | 80292227 | 0.004083148 | 2694 | RASGRF2 | 80256507 | 80525981 | ENSG00000113319 | A-V marker | 201, 203, 206 | Intron 1-2 |
| chr15 | + | 92668229 | 92668230 | 0.004083148 | 2694 | SLCO3A1 | 92396937 | 92709137 | ENSG00000176463 | A-V marker | 201-202 | Intron 5-6 |
| chr15 | + | 92668229 | 92668230 | 0.004083148 | 2694 | SLCO3A1 | 92396937 | 92715665 | ENSG00000176463 | A-V marker | 204 | Intron 5-6 |
| chr15 | + | 92668229 | 92668230 | 0.004083148 | 2694 | SLCO3A1 | 92397564 | 92698499 | ENSG00000176463 | A-V marker | 208 | Intron 3-4 |
| chr15 | + | 92668229 | 92668230 | 0.004083148 | 2694 | SLCO3A1 | 92397564 | 92706228 | ENSG00000176463 | A-V marker | 209 | Intron 5-6 |
| chr4 | - | 186522181 | 186522182 | 0.004083148 | 2694 | SORBS2 | 186506597 | 186578123 | ENST00000355634 | A-V marker | 201, 230 | Intron 18-19 |
| chr4 | - | 186744858 | 186744859 | 0.004083148 | 2694 | SORBS2 | 186506597 | 186877870 | ENST00000355634 | A-V marker | 201, 218, 226, 219, 208, 249, 246 | Intron 1-2 |
| chr4 | - | 186522181 | 186522182 | 0.004083148 | 2694 | SORBS2 | 186506597 | 186733410 | ENST00000355634 | A-V marker | 209 | Intron 12-13 |
| chr4 | - | 186522181 | 186522182 | 0.004083148 | 2694 | SORBS2 | 186506597 | 186732258 | ENST00000355634 | A-V marker | 202 | Intron 19-20 |
| chr4 | - | 186522181 | 186522182 | 0.004083148 | 2694 | SORBS2 | 186506597 | 186732048 | ENST00000355634 | A-V marker | 265 | Intron 2-3 |
| chr4 | - | 186522181 | 186522182 | 0.004083148 | 2694 | SORBS2 | 186506597 | 186697066 | ENST00000355634 | A-V marker | 203 | Intron 20-21 |
| chr4 | - | 186522181 | 186522182 | 0.004083148 | 2694 | SORBS2 | 186506597 | 186606000 | ENST00000355634 | A-V marker | 221 | Intron 20-21 |
| chr4 | - | 186522181 | 186522182 | 0.004083148 | 2694 | SORBS2 | 186506597 | 186599976 | ENST00000355634 | A-V marker | 204 | Intron 21-22 |
| chr4 | - | 186522181 | 186522182 | 0.004083148 | 2694 | SORBS2 | 186510832 | 186696520 | ENST00000355634 | A-V marker | 206 | Intron 20-21 |
| chr4 | - | 186744858 | 186744859 | 0.004083148 | 2694 | SORBS2 | 186547819 | 186877870 | ENST00000355634 | A-V marker | 256 | Intron 2-3 |
| chr2 | - | 135417821 | 135417822 | 0.004083148 | 819 | TMEM163 | 135213329 | 135476571 | ENSG00000152128 | A-V marker | 201 | Intron 2-3 |

**HUVEC**

| AGO2-clip data | | | | | | Targeted-gene info | | | | | | |
|---|---|---|---|---|---|---|---|---|---|---|---|---|
| **Chromosome** | **Strand** | **chromStart** | **chromEnd** | **FDR** | **count(>=m,k)** | **Gene symbol** | **Gene Start** | **Gene End** | **Ensembl ID** | **Association** | **Variants** | **Region** |
| chr15 | - | 58356100 | 58356101 | 0 | 1688 | ALDH1A2 | 58245621 | 58358121 | ENSG00000128918 | A-V marker | 201,202,204-205,212 | Intron 1-2 |
| chr15 | - | 58356100 | 58356101 | 0 | 1688 | ALDH1A2 | 58245621 | 58571462 | ENSG00000128918 | A-V marker | 203.208 | Intron 2-3 |
| chr7 | + | 69235780 | 69235781 | 0.00450045 | 5555 | AUTS2 | 69063904 | 69755607 | ENSG00000158321 | A-V marker | 201-203,221, 224 | Intron 1-2 |
| chr7 | + | 69235780 | 69235781 | 0.00450045 | 5555 | AUTS2 | 69063904 | 70257885 | ENSG00000158321 | A-V marker | 212 | Intron 2-3 |
| chr7 | + | 70197820 | 70197821 | 0.00450045 | 5555 | AUTS2 | 69063904 | 70257885 | ENSG00000158321 | A-V marker | 201,203, 224 | Intron 6-7 |
| chr21 | + | 30672401 | 30672402 | 0.00450045 | 5555 | BACH1 | 30671219 | 30718469 | ENSG00000156273 | HIF | 201, 11,212 | Intron 1-2 |
| chr21 | + | 30672401 | 30672402 | 0.00450045 | 5555 | BACH1 | 30671736 | 30718469 | ENSG00000156273 | HIF | 209,210, 213 | Intron 2-3 |
| chr8 | - | 27455739 | 27455740 | 0.00450045 | 5555 | CLU | 27454433 | 27468874 | ENSG00000120885 | A-V marker/NO | 201.202 | Exon 9 |
| chr8 | - | 27455739 | 27455740 | 0.00450045 | 5555 | CLU | 27454433 | 27468954 | ENSG00000120885 | A-V marker/NO | 211 | Exon 8 |
| chr8 | - | 27455739 | 27455740 | 0.00450045 | 5555 | CLU | 27454433 | 27469268 | ENSG00000120885 | A-V marker/NO | 208 | Exon 3 |
| chr8 | - | 27455739 | 27455740 | 0.00450045 | 5555 | CLU | 27454433 | 27472328 | ENSG00000120885 | A-V marker/NO | 215 | Exon 8 |
| chr1 | - | 86043848 | 86043849 | 0 | 1688 | DDAH1 | 85784167 | 86044046 | ENSG00000153904 | NO | 202.204 | Exon 1 |
| chr19 | + | 10848640 | 10848641 | 0.00450045 | 5555 | DNM2 | 10823793 | 10942579 | ENSG00000079805 | NO | 202.205 | Intron 1-2 |
| chr19 | + | 10848640 | 10848641 | 0.00450045 | 5555 | DNM2 | 10828728 | 10942586 | ENSG00000079805 | NO | 207 | Intron 1-2 |
| chr19 | + | 10897510 | 10897511 | 0.00879397 | 2388 | DNM2 | 10828728 | 10942586 | ENSG00000079805 | NO | 201.205 | Intron 7-8 |
| chr19 | + | 10897510 | 10897511 | 0.00879397 | 2388 | DNM2 | 10893635 | 10942586 | ENSG00000079805 | NO | 209.218 | Intron 3-4 |
| chr19 | + | 10897510 | 10897511 | 0.00879397 | 2388 | DNM2 | 10823793 | 10942579 | ENSG00000079805 | NO | 210 | Intron 2-3 |
| chr2 | + | 46608746 | 46608747 | 0.00450045 | 5555 | EPAS1 | 46524540 | 46613842 | ENSG00000116016 | HIF | 206 | Exon 2 |
| chr2 | + | 46608746 | 46608747 | 0.00450045 | 5555 | EPAS1 | 46606838 | 46613842 | ENSG00000116016 | HIF | 205 | Exon 1 |
| chr18 | + | 19763043 | 19763044 | 0.00450045 | 5555 | GATA6 | 19749403 | 19782491 | ENSG00000141448 | HIF | 201 | Intron 6-7 |
| chr18 | + | 19763043 | 19763044 | 0.00450045 | 5555 | GATA6 | 19750893 | 19782491 | ENSG00000141448 | HIF | 202 | Intron 5-6 |
| chr3 | + | 40145326 | 40145327 | 0.00450045 | 5555 | MYRIP | 39851302 | 40301811 | ENSG00000170011 | A-V marker | 201,203,205 | Intron 3-4 |
| chr3 | + | 40145326 | 40145327 | 0.00450045 | 5555 | MYRIP | 39851977 | 40204453 | ENSG00000170011 | A-V marker | 207,209,210 | Intron 1-2 |
| chr3 | + | 40145326 | 40145327 | 0.00450045 | 5555 | MYRIP | 40141501 | 40301811 | ENSG00000170011 | A-V marker | 202.206 | Intron 3-4 |
| chr2 | - | 178121060 | 178121061 | 0.00450045 | 5555 | NFE2L2 | 178095030 | 178128617 | ENSG00000116044 | HIF | 201-204,206-208,212 | Intron 1-2 |
| chr2 | - | 178121060 | 178121061 | 0.00450045 | 5555 | NFE2L2 | 178095030 | 178129859 | ENSG00000116044 | HIF | 211 | Intron 4-5 |
| chr2 | - | 178121060 | 178121061 | 0.00450045 | 5555 | NFE2L2 | 178095030 | 178257419 | ENSG00000116044 | HIF | 205.21 | Intron 1-2 |
| chr2 | - | 178121060 | 178121061 | 0.00450045 | 5555 | NFE2L2 | 178097004 | 178128617 | ENSG00000116044 | HIF | 213 | Intron 3-4 |
| chr2 | - | 178121060 | 178121061 | 0.00450045 | 5555 | NFE2L2 | 178098204 | 178128617 | ENSG00000116044 | HIF | 214 | Intron 2-3 |
| chr7 | + | 150700571 | 150700572 | 0 | 1688 | NOS3 | 150688143 | 150711687 | ENSG00000164867 | NO | 201 | Intron 14-15 |
| chr7 | + | 150700571 | 150700572 | 0 | 1688 | NOS3 | 150690840 | 150701023 | ENSG00000164867 | NO | 204.21 | Exon 14 |
| chr2 | + | 206553352 | 206553353 | 0 | 1688 | NRP2 | 206547223 | 206608422 | ENSG00000118257 | A-V marker | 216 | Intron 1-2 |
| chr2 | + | 206553352 | 206553353 | 0 | 1688 | NRP2 | 206547223 | 206641880 | ENSG00000118257 | A-V marker | 206,207,209 | Intron 2-3 |
| chr2 | + | 206553352 | 206553353 | 0 | 1688 | NRP2 | 206547223 | 206662857 | ENSG00000118257 | A-V marker | 201-205,214 | Intron 1-2 |
| chr2 | + | 206619302 | 206619303 | 0 | 1688 | NRP2 | 206547223 | 206662857 | ENSG00000118257 | A-V marker | 201-205 | Intron 12-13 |
| chr2 | + | 206553352 | 206553353 | 0 | 1688 | NRP2 | 206547358 | 206608422 | ENSG00000118257 | A-V marker | 208.211 | Intron 2-3 |
| chrX | + | 70761215 | 70761216 | 0.00450045 | 5555 | OGT | 70752911 | 70765701 | ENSG00000147162 | HIF | 201-203,210,211 | Intron 3-4 |
| chrX | + | 70761215 | 70761216 | 0.00450045 | 5555 | OGT | 70752911 | 70795747 | ENSG00000147162 | HIF | 201,202,204,211 | Intron 4-5 |
| chrX | + | 70765745 | 70765746 | 0.00450045 | 5555 | OGT | 70752911 | 70795747 | ENSG00000147162 | HIF | 210 | Exon 4 |
| chr12 | + | 121669455 | 121669456 | 0.00450045 | 5555 | P2RX4 | 121647663 | 121670479 | ENSG00000135124 | NO | 202,205,210,211 | Intron 8-9 |
| chr12 | + | 121669455 | 121669456 | 0.00450045 | 5555 | P2RX4 | 121647663 | 121671909 | ENSG00000135124 | NO | 203.212 | Intron 9-10 |
| chr5 | + | 31733054 | 31733055 | 0.00450045 | 5555 | PDZD2 | 31639516 | 32111038 | ENSG00000133401 | A-V marker | 202,204,211 | Intron 1-2 |
| chr7 | - | 132169186 | 132169187 | 0.00450045 | 5555 | PLXNA4 | 131808090 | 132261323 | ENSG00000221866 | A-V marker | 201,202 | Intron 3-4 |
| chr7 | - | 132169186 | 132169187 | 0.00450045 | 5555 | PLXNA4 | 132068246 | 132261323 | ENSG00000221866 | A-V marker | 204 | Exon 4 |
| chr10 | + | 89668540 | 89668541 | 0.00450045 | 5555 | PTEN | 89623194 | 89728532 | ENSG00000171862 | HIF | 201 | Intron 2-3 |
| chr2 | - | 11361382 | 11361383 | 0.00450045 | 5555 | ROCK2 | 11321777 | 11484711 | ENSG00000134318 | NO | 201,202,209; 203; 207 | Exons 9; 5; 1 |
| chr2 | - | 11414188 | 11414189 | 0.00450045 | 5555 | ROCK2 | 11321777 | 11484711 | ENSG00000134318 | NO | 201,202,206,209 | Intron 3-4 |

| | | | | | | | | | | | | | |
|---|---|---|---|---|---|---|---|---|---|---|---|---|---|
| chr8 | + | 15531296 | 15531297 | 0.00879397 | 2388 | TUSC3 | 15397595 | 15624158 | ENSG00000104723 | A-V marker | 201,202,204,206,209,210,213; 212; 207 | Exon 6; Intron 5-6; Exon 1 |
| chr4 | - | 177697168 | 177697169 | 0.00450045 | 5555 | VEGFC | 177604690 | 177713895 | ENSG00000150630 | HIF | 202 | Intron 1-2 |
| chr14 | - | 69255344 | 69255345 | 0.00450045 | 5555 | ZFP36L1 | 69254371 | 69260631 | ENSG00000185650 | HIF | 201; 202,204 | Exons 3; 2 |
| chr14 | - | 69255609 | 69255610 | 0.00450045 | 5555 | ZFP36L1 | 69254371 | 69260631 | ENSG00000185650 | HIF | 201 | Intron 2-3 |
| chr14 | - | 69255609 | 69255610 | 0.00450045 | 5555 | ZFP36L1 | 69254371 | 69262960 | ENSG00000185650 | HIF | 202.204 | Exon 2 |

**Table S6: List of primer sequences for confirmatory analysis.**

Related to Star Methods "Quantification of miRNA and mRNA levels"

| Target | Primer | Sequence |
|---|---|---|
| miR-21-5p | Forward | 5'-UAGCUUAUCAGACUGAUGUUGA-3' |
| miR-126-3p | Forward | 5'-UCGUACCGUGAGUAAUAAUGCG-3' |
| eNOS | Forward | 5`-CGCCTGATGAGGAGAAGCC-3´ |
| | Reverse | 5`-TCTGTGGTCACCTGAAAC CCT-3´ |
| DDAH1 | Forward | 5`ATGCAACTTTAGATGGCGG AG-3´ |
| | Reverse | 5`-GACCCAATTGCGA TCAGGTTA-3´ |
| Arg-2 | Forward | 5`-TGGCTTGATGAAAAGGCTCTCCAG -3´ |
| | Reverse | 5`-TTGGCAAGACCCACTGA GCGT-3´ |
| SOD1 | Forward | 5`-GGTGTGGCCGATGTGTCTAT-3´ |
| | Reverse | 5`-CCTTTGCCCAAGTCATCTGC-3´ |
| ATP5F1 | Forward | 5`-GCCCTGACAGATTCTCCTATCG-3´ |
| | Reverse | 5`-CAATACCCCTGGACCTAGGAA G-3´ |
| RPLP2 | Forward | 5`-GCGCCAAGGACATCAAGAAG-3` |
| | Reverse | 5`-CCAGCAGGTACACTGGCAA-3` |
